# Gambling habits and Probability Judgements in a Bayesian Task Environment

**DOI:** 10.1007/s10899-024-10339-x

**Published:** 2024-08-27

**Authors:** David L. Dickinson, Parker Reid

**Affiliations:** 1 Department of Economics and CERPA, Appalachian State University, Boone, NC USA; 2 IZA, Bonn, Germany; 3 ESI (Chapman University), Orange, CA USA; 4 Auckland University of Technology, Auckland, Auckland CBD New Zealand

**Keywords:** Gambling, Bayes Rule, Probability Judgements, Cognitive Reflection

## Abstract

Little is known about how gamblers form probability assessments. This paper reports on a preregistered study that administered an incentivized Bayesian choice task to *n* = 465 self-reported gamblers and non-gamblers. The task elicits subjective probability assessments and allows one to estimate the degree to which distinct information sources are weighted in forming probability assessments. Our data failed to support our main hypotheses that experienced online gamblers would be more accurate than non-gamblers in estimating probabilities, that gamblers experienced in games of skill (e.g., poker) would be more accurate than gamblers experienced only in non-skill games (e.g., slots), that accuracy would differ by sex, or that information sources would be weighted differently across different participant groups. Exploratory analysis, however, revealed that gambling frequency predicted lower Bayesian accuracy, while cognitive reflection predicted higher accuracy. The decline in accuracy linked to self-reported gambling frequency was stronger for female participants. Decision modeling estimated a decreased weight place on new evidence (over base rate odds) for those participant groups who showed decreased accuracy, which suggests that a proper incorporation of new information is important for probability assessments. Our results link online gambling frequency to worse performance in the critical probability assessment skills that should benefit gambling success (i.e., in skill-based games). Additional research is needed to better understand the mechanism linking reported gambling frequency to probability assessment accuracy.

**Supplementary Information:**

The online version contains supplementary material available at 10.1007/s10899-024-10339-x.

## Introduction

Decision-making under uncertainty often requires updating of probability assessments given new evidence or information. A disproportionate focus on one source of information over another can lead to inaccurate assessments of uncertainty, which can impact outcomes in many decision domains. This paper examines how self-reported gambling habits affect decision-making in a controlled laboratory task targeting probability assessment skills that can be useful when gambling. We followed a pre-registered design, data collection, and analysis plan, and we contributed additional exploratory analysis as well.

This study administered a choice task that allows one to examine the accuracy of subjective probability assessments against the objective outcome probabilities calculated using Bayes rule.^1^ Additionally, our empirical strategy can estimate the weight participants placed on the difference sources of information available in the Bayesian task. Our main objective was to test for differences in probability assessments by sex, self-reported gambling experience, and self-reported problem-gambling behaviors. While our preregistered hypotheses were established from previous findings, our data ultimately showed no support for hypothesized differences in assessment accuracy or information source weighting among these groups. Rather, exploratory analysis revealed two characteristics that robustly predicted worse probability assessments in the Bayesian task: more frequent gambling, and lower levels of cognitive reflection. We conduct analysis that further links predicted decreases in Bayesian accuracy in our sample to a decreased weight placed on new information relative to base rate odds. We later discuss implications of these findings.

## Background

Bayesian updating has been extensively studied and it represents a building block decision environment of long-standing interest to psychologists (Phillips & Edwards, 1966) and economists (Grether, 1980), among others. Bayes rule suggests a precise way to use new information to update a probability estimate, but cognitive short-cuts may often be employed as an alternative (Kahneman & Tversky, 1973; Tversky & Kahneman, 1973). While some argue that individual differences in intuitive versus deliberative decision styles are not so important in risky choice (Steingroever et al., 2018), a larger body of literature connects Bayesian updating to one’s ability to engage in more deliberative thinking (e.g., Dickinson & Drummond, 2008; Barash et al., 2019; Dickinson & McElroy, 2019).

There has been limited attention on how gamblers evaluate probabilities, which has focused mostly on regular or problem gambler samples (Lim et al., 2015; Cowley et al., 2015). Probability judgments are ubiquitous in the world of gambling, and the average gambler loses money (Stetzka & Winter, 2021). Some evidence suggests that features of certain gambling games may exist to deliberately bias one’s judgment of the games’ expected payoff (Walker et al., 2023). Another view is that gamblers fall prey to decision biases related to probability assessments (Newstead et al., 1992; Pennycook et al., 2015) in the direction consistent with a decreased reliance on deliberative thinking. One’s ability to more accurately update probabilities should pay dividends in the world of gambling, and so the question of whether gambling experience or gambler characteristics can predict probability judgments or one’s approach to probability updating is of interest.

Previous studies have shown that more impulsive regular gamblers exhibited diminished use of an optimal (Bayesian-derived) probability estimate, and they also displayed slower learning rates compared to less impulsive gamblers (Lim et al., 2015). While their sample was small (*n*=87 participants) and subgroups were not examined, such evidence is consistent with the viewpoint that gamblers use relatively less deliberate thought processes in updating beliefs. Ligneul et al. (2013) compared pathological gamblers and matched healthy controls using a risky choice paradigm that allowed them to estimate probability weighting function. They showed that pathological gamblers more likely distorted probabilities, which suggests an increased overweighting of low probabilities and an underweighting of high probabilities (e.g., see Kahneman & Tversky, 1984).

Other research showed that problem gamblers exhibited “illusion of control” behaviors, where they evaluated their gambling streaks primarily based on their largest win, rather than on their largest loss (Cowley et al., 2015). There may also be important sex-related differences regarding gambling behaviors—males were observed to take more risks, to partake in riskier games, and they tended to have more problems with gambling than females (Wong et al., 2013).

We contribute to the literature by bringing new data to this question of how gambling experience and behaviors may predict performance and belief formation in information updating environments. We recruited roughly equal samples of male and female participants from an online platform who reported either experience or no experience with online gambling games. Participants self-reported gambling frequency and problem gambling behaviors. Participants were then administered an incentivized probability assessment task that elicited one’s beliefs regarding the likelihood of an uncertain event. The validated task systematically varied base rate probabilities and sample evidence information across several trials. This task paradigm allows us to examine belief accuracy and estimate the extent to which individuals value or “weight” (or distort) base rate probabilities and new information in belief updating.

### Hypotheses

Our pre-registered hypotheses were based on the existing research that shows decision-making differences among participant subgroups. Some hypotheses focused on the accuracy of probability assessments relative to the objective Bayesian probability. A second set of hypotheses focused on how decision makers weight both sources of information in the task environment (i.e., differential weighting of information sources, or not weighting the information as fully as Bayes rule would predict). While Bayes rule establishes the precise way in which new sample evidence should combine with base rate probabilities to generate the updated probability estimate (Bayes, 1958), the hypotheses largely follow from empirical findings that have documented how real-world decision makers deviate from the predictions of Bayes rule. For example, Holt and Smith (2009) showed new information was fully weighted in accordance with Bayes rule, but probability weighting suggesting an over-weighting of low probability base rates and under-weighting of high probability base rates.

Person-specific characteristics have also been shown to impact the assessment of probabilities. For example, past research indicates that males tend to overestimate the perceived odds in a gambling environment, and they exhibit different behaviors than females (Wong et al., 2013). Thus, we anticipated that there would be a significant difference in Bayesian accuracy and information source weighting by sex in our data. Because past research connects impulsive or problem gambling behaviors with poor performance (Lim et al., 2015; Cowley et al., 2015) in Bayesian environments, we also hypothesized accuracy and information source weight differences between problem and non-problem gamblers. We also considered that experience with skill-based gambling games (e.g., poker or sports betting) would likely imply a better Bayesian decision maker as compared to a gambler who only reported experience with games of chance (e.g., slots or Pachinko). Here, we note that previous research makes a distinction between games of skill versus luck (e.g., Chantal & Vallerand, 1996; Getty et al., 2018), because games of skill involve feedback learning that is essentially a Bayesian updating exercise aimed at more accurately assessing game related probabilities. Below are the full set of our preregistered hypotheses, which we divide into hypotheses related to assessment accuracy versus decision weighting.^2^

### Accuracy Hypotheses

- *Hypothesis 1: Bayesian accuracy will differ by sex*
- *Hypothesis 2: Non-problem gamblers will make more accurate probability assessments than problem gamblers*
- *Hypothesis 3: Non-problem gamblers experienced in skilled gambling games will make more accurate assessments than those experienced only in unskilled games*

### Information Source Weight Hypotheses

- *Hypothesis 4a: Participants will respond fully to sample evidence information*
- *Hypothesis 4b: Participants will underweight low and overweight high probability base rates*
- *Hypothesis 5a: Information source weights will differ between those experienced in games of skill versus those experienced only in non-skill games (or non-gamblers)*
- *Hypothesis 5b: Information source weights will differ between those scoring higher on problem gambling behavior versus others (nongamblers or non-problem gamblers)*
- *Hypothesis 6: Problem gamblers will display more severe base rate weighting bias than non-problem gamblers or non-gamblers.*

Though we did not preregister a hypothesis regarding frequency of gambling and task performance, there is a basis for exploratory analysis of the importance of self-reported gambling frequency. Frequent gamblers tend to be overconfident in their abilities to predict odds and this leads them to typically perform more poorly than others (Cowley et al., 2015). And, almost by definition an impulsive or problem gambler will be a more frequent gambler. Thus, we further examine the importance of gambling frequency independent of one’s problem-gambler status in the exploratory analysis we conducted.

## Methods

### Survey and sample screening details

The methods used were preregistered on the Open Science Framework (https://osf.io/zjsg7) to establish hypotheses, sample sizes, variable specifications, and analysis plans. All methods for data collection were carried out in accordance with the US Federal Policy for the Protection of Human Subjects, and our procedures were approved by the human subjects review board at the author’s academic institution.

Our sample was recruited from the Prolific platform (Palan & Schitter, 2018; Peer et al., 2022), which integrates seamlessly with popular survey software platforms to administer surveys and decision-making studies. Importantly, Prolific allows researchers to recruit custom research participant samples based on criteria within the participant’s Prolific profile. Our inclusion criteria for this study were: young adults located in the U.S. and the U.K. who were between 21 and 48 years of age; self-reported experience or lack of experience with one or more (or none) of the games from of a list of popular online gambling games—we recruited half our sample from among those reporting experience and half reporting no experience with any of the online gambling games listed. We limited our study to participants between the ages of 21 and 48, as the age of 21 is the legal minimum age to gamble in most states in the United States, and research shows that cognitive decline is already evident in middle age (45-49 years) (Singh-Manoux et al., 2012). Thus, our sample was chosen to eliminate any potential confound between age-related cognitive decline and performance in our Bayesian task, which would classify as an executive function task.

Our planned sample size was partly based on available funds, but we also conducted an a priori power analysis using G*Power 3.1.9.4. Here, we found that a planned sample of n=400 would have sufficient power (power = .80 for behavioral research) to detect a small effect size (*f* ^2^ = .02) for a single regression coefficient in a multiple regression with up to 6 co-variates (e.g., age, sex, gambling experience), assuming an α = .05 error probability. A medium-small effect size (*f* ^2^ = .065) is detectible with a sample size of n=100, which means we may also conduct sufficiently powered analysis of decision model estimates on separate subsamples (e.g., females with gambling experience, males with no gambling experience).

### The Bayesian decision task

**Fig. 1:**
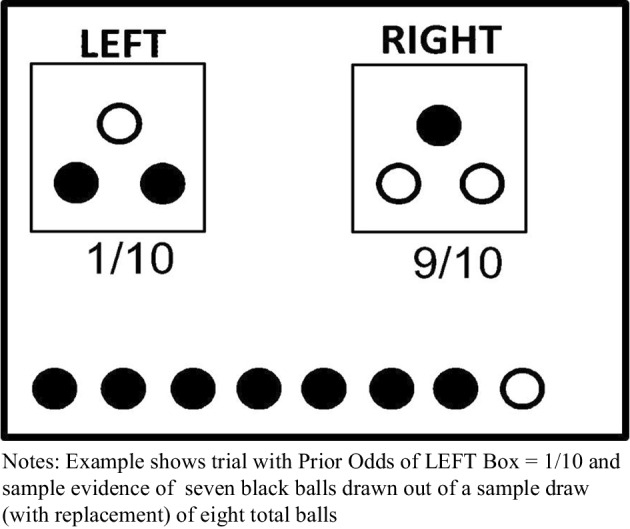
Sample Bayes task stimulus

Our incentivized decision task is a modification of the Grether’s design (Grether, 1980) that has been adopted by others in recent literature (Dickinson and Garbuio, 2021).^3^ For the decision task, there are two boxes each populated with three balls. As shown in Figure 1, the LEFT box has two black and one white ball. Either the LEFT or RIGHT box will be selected in a trial. The participant is not told which box is selected for the current trail, but she is presented with two sources of information with which to form beliefs regarding which box was selected: the base rate or “prior odds” of either box being selected, and the results from drawing eight balls with replacement from the chosen (but hidden) box. The prior odds were represented as the chances out of ten that either box would be selected, ex ante, and this can be considered the initial information for that stimulus (trial). The results of the eight-ball sample draw can be considered the new evidence presented to the participant for that stimulus. As shown in Figure 1, the stimulus image offered a visually concise way to present this information to the participant, and the task varied the prior odds and/or new evidence across twenty decision trials (see shaded cells in Table 1).

**Table 1:**
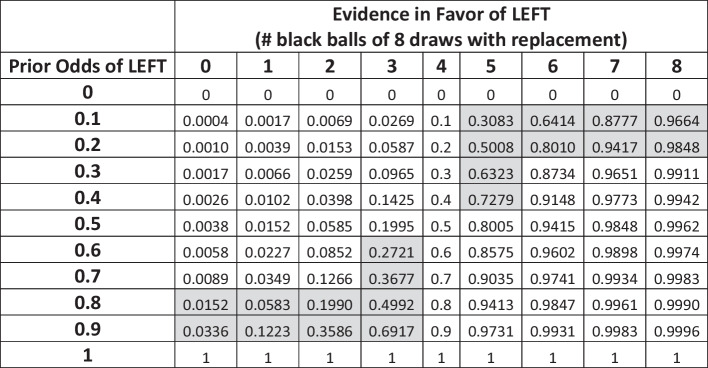
Bayesian probabilities by odd-evidence combination (highlighted cells show those combinations administered to participants in the study)

Notes: Bayes probabilities of the LEFT box being used were calculated using Bayes rule:

$$P\left(Left|X\right)=\frac{P\left(X|Left\right)P(Left)}{P\left(X|Left\right)P\left(Left\right)+P\left(X|Right\right)P(Right)}$$

In each trial the participant is asked to give a subjective assessment of how likely it was that the LEFT box had been used or selected in that trial (i.e., the “chances out of 100” that the LEFT box was used), which we call “*Left Assess*” ∈ [0, 100]. The elicitation of a precise subjective probability estimate, rather than a dichotomous response of which box was more likely (see Grether, 1992), provides more rich data to the extent that participants are incentivized to provide truthful subjective probability estimates. To this end, we followed Holt and Smith (2000) and used a Becker-DeGroot-Marshak type cross-over scoring procedure. Here, participants maximize the chance of a bonus payment in each trial when providing one’s true subjective probability estimate (see Experiment Instructions in Appendix B). Because the incentivization procedure is somewhat complicated, participants are reminded at the end of the instructions that they would maximize their expected bonus payment in each trial “….by responding with your true belief of how likely you think the LEFT box was selected, given the available information!”

Table 1 shows the objective conditional probabilities, which are calculated by Bayes rule, for all possible combinations of prior odds and evidence. For this study, each participant was administered the 20 highlighted combinations in randomized order, which focused on situations where the odds and evidence favored opposite boxes. For example, in one trial the prior odds of the LEFT box were 1/10 (indicating the RIGHT box is more likely to be used) but the number of black balls drawn in the sample evidence was 7 out of 8 (i.e., a draw more likely if the LEFT box rather than the RIGHT box is used). Thus, the odds and evidence point to opposite more likely boxes and make the Bayesian probability assessment task more challenging than if odds and evidence favored the same box.

### Dependent Variables and Estimation Strategy

For the analysis of the accuracy of one’s subjective probability assessments in each trial, the dependent variable *Accuracy* was defined (as in Dickinson and Garbuio, 2021) by the absolute difference between *Left Assess* and the True Bayes Probability, ∈ [0,1]:

$$Accuracy=1-\left|\left(Left\;Assess/100\right)-True\;Bayesian\;Probability\right|\in [\text{0,1}]$$

For participant-level analysis (i.e., one summary accuracy observation per participant), *Average Accuracy* across all 20 trials was used as the dependent variable for both nonparametric tests and ordinary least squares regression models. Individual trial-level analysis used *Accuracy* as the dependent variable in linear regression models that included robust standard errors clustered on the participant (i.e., 20 observations per participant). Hypotheses 1-3 are examined below using both participant-level and then trial-level data.

Decision model estimations required the trial-level observations (20 observations per participant). The estimation strategy for the decision model followed the approach in Holt and Smith (2000) (see also Wu and Gonzalez, 1996).^4^ Here, one holds a subjective belief regarding the probability that the LEFT box was used in a trial, which we call *Belief*_*LEFT,*_ and the key dependent variable used was the log-odds ratio of one’s belief that the LEFT versus RIGHT box was used, $ln\left(\frac{{Belief}_{LEFT}}{1-{Belief}_{LEFT}}\right)$. The empirical log-odds version of Bayes rule defines *Belief*_*LEFT*_ as a function of the log of the prior odds ratio (i.e., the “prior” odds) and the log of the likelihood ratio of the given sample draw (i.e., the “evidence”, which is the likelihood of the sample, *S*, given the LEFT box was used divided by the likelihood of the sample given the RIGHT box was used).

4 $$ln\left(\frac{{Belief}_{LEFT}}{1-{Belief}_{LEFT}}\right)=\alpha +{\gamma }_{1}ln\left(\frac{{Prob}_{LEFT}}{\left(1-{Prob}_{LEFT}\right)}\right)\bullet {\gamma }_{2}ln\left(\frac{Prob(S|LEFT)}{Prob\left(S|RIGHT\right)}\right)$$

In other words, the subjective odds ratio favoring the LEFT box is a function of the prior odds of the LEFT box and the evidence that favors the LEFT box. Additional control variables supplement this baseline specification in equation (4), and the model is estimated via linear regression with robust standard errors clustered on the participant.

## Independent Variables

Information was collected on participant demographics (e.g., age, sex, US versus UK resident), self-reported gambling habits, and other descriptors of one’s current cognitive state (i.e., sleepiness)^5^ and whether one was a more reflective or intuitive decision maker.

The key independent variables needed to test our hypotheses were as follows: *Female* was an indicator variable denoting the participant’s self-reported sex as assigned at birth. A participant was scored as *Problem Gambling* = 1 if reporting any “yes” response on the NODS-CLiP* Short Problem Gambling Screen (Volberg et al., 2011). Participants also self-reported which, if any, online gambling games they had played from a list of fourteen options, and a participant was coded as *Skill Gambler* = 1 if they reported playing games of skill (i.e., Blackjack, Poker, Video Poker, Race & Sports Book, Virtual Sports Betting (Chantal & Vallerand, 1996; Getty et al., 2018). *Gambling Frequency* was assessed on a 5-option Likert scale from “Never” *(Gambling Frequency =* 0) to “Daily” (*Gambling Frequency =* 4).^6^ Finally, one’s reflective versus intuitive thinking style was assessed using the 6-item Cognitive Reflection Task (Primi et al., 2016), which produced a 0-6 *CRT score* variable, where higher values indicate a more reflective thinker.

## Results

The final sample obtained was n=465 individuals (n=220 who self-reported online gambling experience, n=245 who self-reported no online gambling experience)—14 of those self-reporting gambling experience reported a current gambling frequency of “never” and so were coded as *Nongamblers*. Among these, our sample included n=230 females (n=235 males), and n=90 *Problem gamblers*. A total of n=135 participants resided in the *United States* and n=330 in the United Kingdom. Table 2 shows the summary statistics on key individual-specific control measures that will be used in our analysis.

**Table 2:** Summary statistics by gambling group

Variable	Non-problem Gambler	Problem Gambler	Unskilled Gambler	Skilled Gambler
Age	Mean = 32.285 SD = 7.709	Mean = 32.022 SD = 7.929	Mean = 31.821 SD = 7.790	Mean = 32.925 SD = 7.639
Female = 1	Number (proportion) 195 (52%)	Number (proportion) 35 (39%)	Number (proportion) 154 (53%)	Number (proportion) 76 (44%)
U.K. = 1 (vs. USA)	Number (proportion) 265 (71%)	Number (proportion) 65 (72%)	Number (proportion) 221 (76%)	Number (proportion) 109 (63%)
Gambling Frequency*	Mean = 0.576 SD = 0.942	Mean = 1.800 SD = 1.104	Mean = 0.216 SD = 0.597	Mean = 1.810 SD = 0.988
CRT score	Mean = 3.325 SD = 2.121	Mean = 3.356 SD = 1.97	Mean = 3.289 SD = 2.180	Mean = 3.379 SD = 1.937
Total participants	375	90	291	174

Gambling Frequency was self-reported by participants, on a scale of 0-4: 0 (never gamble); 1 (less than once a month); 2 (once or twice a month); 3 (once or twice a week); 4 (daily). For example, a score of 1.800 would indicate that identified Problem gamblers, on average, report current gambling between less than once a month and once or twice a month

### Hypotheses 1-3

The first set of hypotheses focus on the accuracy of one’s probability assessments, relative to Bayes rule. Table 3 shows first evidence of the *lack* of support for hypotheses 1-3. T-tests on the relevant pairwise subsamples highlight the lack of difference in *Average Accuracy* across the 20-trials. Recall also that tests of Hypotheses 2 and 3 require considering only the subsample of data on non-problem gamblers (Hypothesis 2) or the subsample of gamblers (Hypothesis 3), whereas the entire sample of gamblers and non-gamblers is used to evaluate Hypothesis 1. The bottom of Table 3 also shows some initial evidence from *Average Accuracy* linking more frequent gambling with reduced Bayesian accuracy. Table 4 shows results from OLS regressions of *Average Accuracy* on participant characteristics. The Table highlights that while one’s identification as a *Skill Gambler* appears to *negatively* predict Bayesian accuracy, the result is spurious and due to a high correlation (rho = .710) with self-reported *Gambling Frequency*. We discuss the importance of *Gambling Frequency* later.

**Table 3:** Mean *Average Accuracy* and hypothesis tests

Test Variable: *Average Accuracy*	H1 test		H2 test		H3 test	
	Males (n=235)	Females (n=230)	Skill-Game (non-problem) Gamblers (n=99)	NonSkill-Game (non-problem) Gamblers (n=30)	Problem Gamblers (n-77)	Non-Problem Gamblers (n=129)
mean	.727	.709	.689	.690	.693	.689
t-stat (p-value)	1.2695 (p=.205)		.021 (p=.984)		-.160 (p=.873)	
Exploratory--comparison of *Average Accuracy* by current gambling frequency						
		*Gfreq*=0 (n=259)	*Gfreq*=1 (n=93)	*Gfreq*=2 (n=62)	*Gfreq*=3 (n=43)	*Gfreq*=4 (n=8)
	Mean	.740	.716	.674	.670	.634
	St. dev	.144	.158	.163	.166	(.211)

*SkillGame* gambler t-test used the subset of participants who reported current gambling frequency greater than zero. A similar test did not reveal any statistically significant difference in *Accuracy* between nonproblem gamblers who *exclusively* played skill-games compared to those who played a mix of skill games and games of chance, but our data set only includes n=12 current gambler participants who reported exclusively playing gambling games of skill. “*Gfreq*” = *Gambling Frequency* ∈ [0,4], which describes self-reported *current* gambling frequency (0,1,2,3,4 indicates responses of “never”, “less than once a month”, “once or twice a month”, “once or twice a week”, or “daily”. Nongambler participants were scored as *Gfreq* = 0

**Table 4:** *Average Accuracy* by sex, *Skill Gambler, Problem Gambler*

Dependent Variable: *Average Accuracy*	(1)	(2)	(3)
Variable	Coef (st. error)	Coef (st. error)	Coef (st. error)
Constant	.747 (.012)**	.778 (.034)**	.676 (.037)**
Female (=1)	-.022 (.014)	-.025 (.015)	-.004 (.015)
*Skill Gambler* (=1)	-.046 (.016)**	-.045 (.016)**	-.007 (.021)
*Problem Gambler* (=1)	-.002 (.019)	-.003 (.020)	.010 (.020)
Age	---	-.001 (.001)	-.0003 (.001)
USA (=1)	---	-.0001 (.017)	.003 (.016)
*Average Response Time*	---	---	.002 (.001)**
*CRT score* ∈ [0,6]	---	---	.015 (.003)**
Gambling Frequency ∈ [0,4]	---	---	-.025 (.010)**
R-squared	.0248	.0268	.1004

*p < .05, **p < 01 for the 1-tailed test of a pre-registered one-sided hypothesis (otherwise, *p-value* is for the 2-tailed test). *N*=465 observations (participants). Results are ordinary least squares estimates. The significant coefficient estimates on the variable *Skill Gambler* in models (1) and (2) is opposite the preregistered hypothesis. This finding is not present once controlling for one’s frequency of gambling (i.e., *Gambling Frequency* controls for those who report more frequent gambling. which spuriously relates to one being more likely to have reported playing an online gambling game of skill—the simple correlation between *Gambling Frequency* and *Skill Gambler* is .710).

Tables 5, 6, 7 properly test Hypotheses 1-3 using the panel nature of the data by regressing trial-level *Accuracy* on the key indicator variables and participant characteristics. Across models (1), (2), and (3) we successively add additional control variables. The sample size differences across Tables 5, 6, 7 reflect the need to use the full sample to test Hypothesis 1, but subsets of the data to test Hypotheses 2 and 3. We focus on the following binary indicator variables to test our hypotheses: *Female* in Table 5 (testing Hypothesis 1), *Skill Gambler* in Table 6 (testing Hypothesis 2), and *Problem Gambler* in Table 7 (testing Hypothesis 3). Coefficient estimates on these variables are all statistically insignificantly different from zero across all specifications, which supports rejecting Hypotheses 1-3. In fact, we find robust support in Tables 5, 6, 7 that only two variables predict one’s *Accuracy*: *Gambling Frequency* predicts lower *Accuracy*, while *CRT Score* predicts higher *Accuracy.*

**Table 5:** Hypothesis 1 test (Accuracy by sex)—panel data estimates

Dependent Variable: *Accuracy* (trial level)	(1)	(2)	(3)
Variable	Coef (st. error)	Coef (st. error)	Coef (st. error)
Constant	.727 (.010)**	.720 (.011)**	.635 (.066)**
Female (=1)	-.018 (.014)	-.018 (.014)	-.010 (.015)
*Trial #*	---	.0003 (.0004)	.0003 (.0004)
*Response Time*	---	.0003 (.0002)	.0002 (.0002)
Age	---	---	-.0001 (.001)
Gambling Frequency ∈ [0,4]	---	---	-.026 (.007)**
*Prior Week Sleep Level*	---	---	.006 (.006)
*Karolinska sleepiness*	---	---	.003 (.004)
*CRT score* ∈ [0,6]	---	---	.016 (.003)**
R-squared	.0013	.0020	.0339

*p < .05, **p < 01 for the 1-tailed test of a pre-registered one-sided hypothesis (otherwise, *p-value* is for the 2-tailed test). *N*=9300 observations. Linear regression estimates with robust standard errors adjusted for clustering at the participant level (*n*=465 clusters)

**Table 6:** Hypothesis 2 test—Among non-problem gamblers (n=129), those with skill-game experience will make more accurate probability assessments than those with only non-skill-game experience

Dependent Variable: *Accuracy* (trial level)	(1)	(2)	(3)
Variable	Coef (st. error)	Coef (st. error)	Coef (st. error)
Constant	.690 (.031)**	.692 (.033)**	.612 (.134)**
*Skill Gambler* (=1)	-.001 (.035)	-.001 (.035)	-.028 (.037)
*Trial #*	---	-.001 (.001)	-.001 (.001)
*Response Time*	---	.0005 (.0003)	.001 (.0003)
Age	---	---	-.002 (.002)
Female (=1)			-.033 (.030)
Gambling Frequency ∈ [0,4]	---	---	-.039 (.017)*
*Prior Week Sleep Level*	---	---	.023 (.012)
*Karolinska sleepiness*	---	---	.003 (.008)
*CRT score* ∈ [0,6]	---	---	.021 (.006)**
R-squared	.0000	.0016	.0593

*p < .05, **p < 01 for the 1-tailed test of a pre-registered one-sided hypothesis (otherwise, *p-value* is for the 2-tailed test). *N*=2580 observations Linear regression estimates with robust standard errors adjusted for clustering at the participant level (n=129 clusters). Skill-games were considered to be the following: blackjack, poker, sports betting. Non-skill-games were: slots, baccarat, craps, roulette

**Table 7:** Hypothesis 3 test—Among gamblers (*n* = 206), non-problem Gamblers will make more accurate probability assessments than problem gamblers

Dependent Variable: *Accuracy* (trial level)	(1)	(2)	(3)
Variable	Coef (st. error)	Coef (st. error)	Coef (st. error)
Constant	.689 (.015)**	.686 (.017)**	.638 (.099)**
*Problem Gambler* (=1)	.004 (.023)	.004 (.023)	.015 (.024)
*Trial #*	---	.0001 (.001)	.0001 (.001)
*Response Time*	---	.0002 (.0002)	.0001 (.0002)
Age	---	---	-.002 (.002)
Female (=1)			-.029 (.024)
Gambling Frequency ∈ [0,4]	---	---	-.032 (.014)*
*Prior Week Sleep Level*	---	---	.011 (.010)
*Karolinska sleepiness*	---	---	.006 (.006)
*CRT score* ∈ [0,6]	---	---	.022 (.005)**
R-squared	.0000	.0003	.0484

*p < .05, **p < 01 for the 1-tailed test of a pre-registered one-sided hypothesis (otherwise, *p-value* is for the 2-tailed test). *N*=4120 observations. Linear regression estimates with robust standard errors adjusted for clustering at the participant level (n=206 clusters). Skill-games were considered to be the following: blackjack, poker, sports betting. Non-skill-games were: slots, baccarat, craps, roulette

### Hypotheses 4-6

We next turn our attention to an examination of the formation of subjective probability estimates. For these tests, panel estimations were performed on the trial-level data. Hypotheses 4a and 4b are a test of whether, in the baseline specification shown in equation (4) above, ${\gamma }_{1}$=1 (Hypothesis 4a) and whether ${\gamma }_{1}$<1 (Hypothesis 4b). Table 8, column (1), shows the results for the baseline specification, while results in columns (2) and (3) add additional control variables. Across all specifications, the data reject Hypothesis 4a in favor of more *conservative* Bayesian updating (e.g., Phillips and Edwards, 1966). The data support Hypothesis 4b in that we always reject the null hypothesis test that ${\gamma }_{1}$=1 in Table 8.

**Table 8:** Hypothesis 4a and 4b tests (Modeling subjective belief formation)

Dependent Variable: *Ln(Subjective Odds ratio)*_*Left*_	(1)	(2)	(3)
Variable	Coef (st. error)	Coef (st. error)	Coef (st. error)
Constant	.026 (.020)	-.0003 (.042)	-.225 (.155)
*Ln(Prior Odds ratio)*_*Left*_	.324 (.029)**	.324 (.029)**	.324 (.029)**
*Ln(Likelihood ratio)*_*Left*_	.291 (.015)**	.291 (.015)**	.291 (.015)**
*Trial #*	---	.004 (.004)	.004 (.004)
*Response Time*	---	-.001 (.001)	-.001 (.001)
Age	---	---	.003 (.003)
Female (=1)	---	---	.022 (.040)
Gambling Frequency ∈ [0,4]	---	---	-.018 (.019)
*Prior Week Sleep Level*	---	---	.030 (.015)*
*Karolinska sleepiness*	---	---	-.009 (.011)
*CRT score* ∈ [0,6]	---	---	-.019 (.009)*
R-squared	.090	.090	.091

*p < .05, **p < 01 for the 1-tailed test of a pre-registered one-sided hypothesis (otherwise, *p-value* is for the 2-tailed test). *N*=9300 observations. Linear regression estimates with robust standard errors adjusted for clustering at the participant level (*n*=465 clusters)

Table 9 shows results of the test of Hypothesis 5a that *Skill Gamblers* will weight information sources differently than others. For this Hypothesis 5a test, two variables are added that interact the *Skill Gambler* indicator variable with the *ln(PriorOdds ratio)*_*left*_ and with *ln(Likelihood ratio)*_*left*_. The estimation results indicate that someone who self-reported experience with skill-based games places *less* weight on the sample evidence compared to one who did not report experience with skill-game gambling (this includes non-gamblers). This would support Hypothesis 5a, but further analysis seems to reveal that this result is an artifact of the connection between *Skill Gambler* and *Gambling Frequency*.^7^Appendix Table A2 highlights that re-estimation of the models in Table 9 to include interactions terms between *Skill Gambler, Gambling Frequency,* and each of the two information sources leads to statistically insignificant coefficient estimates on the *Skill Gambler* interaction term with *ln(Likelihood ratio)*_*left*_ . In its place, the interaction between Gambling Frequency * ln(Likelihood ratio)_*left*_ is statistically significant and negative. Exploratory analysis below will further examine the importance of *Gambling Frequency* in our data.^8^

**Table 9:** Hypothesis 5a test—Skill-game experience and subjective belief formation

Dependent Variable: *Ln(Subjective Odds ratio)*_*Left*_	(1)	(2)	(3)
Variable	Coef (st. error)	Coef (st. error)	Coef (st. error)
Constant	.044 (.025)	.020 (.045)	-.202 (.155)
*Ln(Prior Odds ratio)*_*Left*_	.328 (.035)**	.328 (.035)**	.328 (.035)**
*Ln(Likelihood ratio)*_*Left*_	.326 (.018)**	.325 (.018)**	.325 (.018)**
*Skill Gambler* (=1)	-.047 (.043)	-.048 (.043)	-.045 (.051)
Skill Gambler * *Ln(Prior Odds ratio)*_*Left*_	-.010 (.061)	-.010 (.061)	-.010 (.061)
Skill Gambler * *Ln(Likelihood ratio)*_*Left*_	-.092 (.033)**	-.091 (.033)**	-.092 (.033)**
*Trial #*	---	.004 (.003)	.004 (.003)
*Response Time*	---	-.001 (.001)	-.001 (.001)
Age	---	---	.003 (.003)
Female (=1)	---	---	.023 (.040)
Gambling Frequency ∈ [0,4]	---	---	-.004 (.024)
*Prior Week Sleep Level*	---	---	.030 (.015)*
*Karolinska sleepiness*	---	---	-.010 (.010)
*CRT score* ∈ [0,6]	---	---	-.019 (.009)*
R-squared	.096	.096	.097

*p < .05, **p < 01 for the 1-tailed test of a pre-registered one-sided hypothesis (otherwise, *p-value* is for the 2-tailed test). *N*=9300 observations. Linear regression estimates with robust standard errors adjusted for clustering at the participant level (*n*=465 clusters)

Table 10 results show tests of Hypotheses 5b and 6, which focused on the subset of *Problem Gamblers*. Interaction terms were added to the baseline specification to perform the statistical tests of these hypotheses, and it is apparent across all models (1)-(3) of Table 10 that the data fail to support H5b and H6—*Problem Gamblers* weighted the information sources no differently than non-problem gamblers or non-gamblers. Overall, we find little support for our preregistered hypotheses, other than evidence for the overweighting of low and underweighting of high prior odds. Regarding the use of new information, the data are consistent with conservative but not optimal Bayesian updating in all participant types.

**Table 10:** Hypothesis 5b and 6 tests—Problem-gamblers and subjective belief formation

Dependent Variable: *Ln(Subjective Odds ratio)*_*Left*_	(1)	(2)	(3)
Variable	Coef (st. error)	Coef (st. error)	Coef (st. error)
Constant	.029 (.022)	.002 (.043)	-.236 (.157)
*Ln(Prior Odds ratio)*_*Left*_	.322 (.031)**	.322 (.031)**	.322 (.031)**
*Ln(Likelihood ratio)*_*Left*_	.298 (.017)**	.298 (.017)**	.298 (.017)**
*Problem Gambler* (=1)	-.012 (.059)	-.010 (.058)	.026 (.058)
Problem Gambler * *Ln(Prior Odds ratio)*_*Left*_	.009 (.079)	.011 (.079)	.011 (.079)
Problem Gambler * *Ln(Likelihood ratio)*_*Left*_	-.037 (.042)	-.035 (.042)	-.035 (.042)
*Trial #*	---	.004 (.003)	.004 (.003)
*Response Time*	---	-.001 (.001)	-.001 (.001)
Age	---	---	.004 (.003)
Female (=1)	---	---	.024 (.040)
Gambling Frequency ∈ [0,4]	---	---	-.022 (.019)
*Prior Week Sleep Level*	---	---	.031 (.015)*
*Karolinska sleepiness*	---	---	-.008 (.011)
*CRT score* ∈ [0,6]	---	---	-.019 (.009)*
R-squared	.091	.091	092

*p < .05, **p < 01 for the 1-tailed test of a pre-registered one-sided hypothesis (otherwise, *p-value* is for the 2-tailed test). *N*=9300 observations. Linear regression estimates with robust standard errors adjusted for clustering at the participant level (*n*=465 clusters)

## Exploratory Analysis

We also report exploratory findings of hypotheses that were not preregistered but were nevertheless of interest. In conducting our preregistered hypotheses tests, it became apparent that two characteristics robustly predicted Bayesian accuracy in our incentivized task: *CRT score* (a measure of more reflective versus automatic thinking) and self-reported *Gambling Frequency*. As such, we pursued exploratory analysis of our Bayesian decision model specification to examine whether differential decision weights on information sources was predicted by either or both of these participant-specific characteristics.

Table 11 shows results of this exploratory analysis, where the baseline belief formation model is modified to include interactions of the odds and evidence variables with *CRT Score* and *Gambling Frequency*. These models were estimated without the control variables that have largely been insignificant predictors (results are similar with their inclusion and are available on request). Here, models (1)-(3) differ by whether we estimate the model on the full sample or on the subsample of male or female participants. Results in Table 11 again show that participants are conservative Bayesian decision makers who engage in probability weighting as a baseline. Those with higher *CRT Scores*, which would indicate more reflective thinkers, place marginally higher weight on the sample evidence compared to those with lower *CRT Scores*, and this effect is robust in both male and female subsample estimations. *Gambling Frequency* predicts a marginally lower weight placed on sample evidence, and this result is driven by the subsample of female participants.

**Table 11:** Examining the important of current *Gambling Frequency* and *CRT Score* on information source weighting--Exploratory

Dependent Variable: *Ln(Subjective Odds ratio)*_*Left*_	All participants (1)	Males (2)	Females (3)
Variable	Coef (st. error)	Coef (st. error)	Coef (st. error)
Constant	.105 (.041)**	.051 (.061)	.142 (.053)**
*Ln(Prior Odds ratio)*_*Left*_	.305 (.057)**	.328 (.095)**	.285 (.075)**
*Ln(Likelihood ratio)*_*Left*_	.211 (.038)**	.194 (.045)**	.242 (.035)**
*CRT score* ∈ [0,6]	-.020 (.01)*	-.011 (.013)	-.026 (.014)
CRT Score * *Ln(Prior Odds ratio)*_*Left*_	.001 (.013)	.002 (.020)	-.001 (.019)
CRT Score * *Ln(Likelihood ratio)*_*Left*_	.036 (.007)**	.041 (.010)**	.027 (.009)**
*Gambling Frequency* ∈ [0,4]	-.015 (.020)	-.004 (.028)	-.030 (.027)
Gambling Frequency * *Ln(Prior Odds ratio)*_*Left*_	.020 (.030)	.003 (.038)	.043 (.050)
Gambling Frequency * *Ln(Likelihood ratio)*_*Left*_	-.050 (.01)**	-.035 (.018)	-.077 (.018)**
Observations (clusters)	9300 (465)	4700 (235)	4600 (230)
R-squared	.1213	.1397	.1104

*p < .05, **p < 01 for the 1-tailed test of a pre-registered one-sided hypothesis (otherwise, *p-value* is for the 2-tailed test). *N*=9300 observations. Linear regression estimates with robust standard errors adjusted for clustering at the participant level (*n*=465 clusters)

**Fig. 2:**
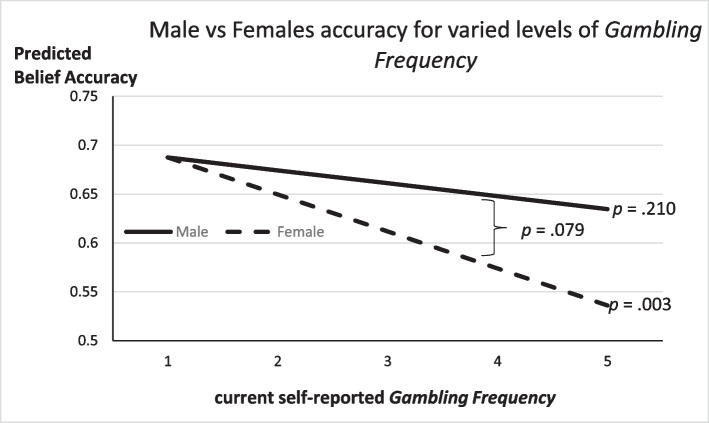
Belief accuracy declines with increased *Gambling Frequency*

**Fig. 3:**
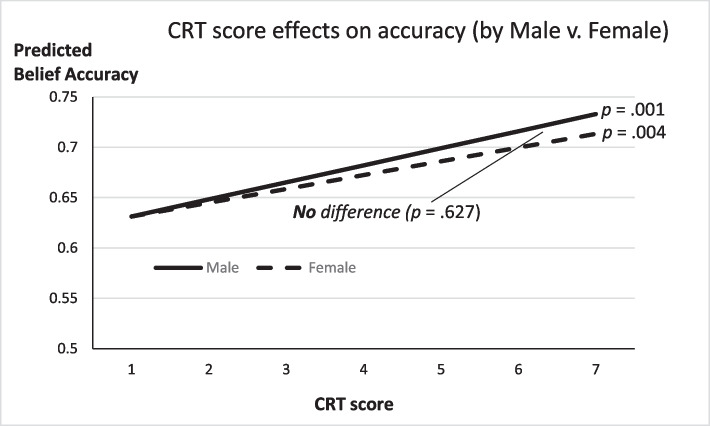
Belief accuracy increases with higher Cognitive Reflection

These Table 11 findings, in conjunction with Tables 4, 5, 6, and 7 results, suggest a mechanism connecting Bayesian accuracy to weighting the evidence more fully. That is, *CRT Score* predicts more accurate Bayesian choices and is also linked to increased weight placed on sample evidence.^9^ And, *Gambling Frequency* is found to reduce Bayesian accuracy but is also linked to a reduced weight on sample evidence in the data, which may also differ by participant sex. A final exploratory analysis estimated the *Accuracy* model (3) from Table 3 and included interaction terms for Female * Gambling Frequency and Female * CRT Score. The results are summarized in Figure 2 and 3, and full results behind these both figures are in the Appendix Table A4. Figure 2 shows that the predicted decline in *Accuracy* among more frequent gamblers is marginally more severe among female participants. This is consistent again with the Table 11 finding that the marginally lower weight placed on sample evidence by female participants corresponds to a greater decay in *Accuracy* for more frequent female gamblers. Figure 3 highlights that the Table 11 result showing that more reflective thinkers, male or female, place marginally more weight on sample evidence is consistent with a significant increase in *Accuracy* for higher *CRT Score* participants regardless of sex.

## Discussion

We set out to test a set of preregistered hypotheses derived from Bayes rule and previous empirical findings, but the data mostly failed to support those hypotheses. Rather, exploratory analysis highlighted that future research may wish to focus on the importance of new information and Bayesian accuracy, and sex differences in probability assessments. The most robust and consistent finding from the exploratory analysis was that more reflective thinkers tend to pay additional attention to new sample evidence in forming beliefs regarding uncertainty, which then improves accuracy in making probability assessments. A second exploratory finding of note was that more frequent gamblers did surprisingly worse in the probability assessments. This result was significant only among female participants and can be linked to a decreased weight placed on new evidence forming beliefs.

It is worth noting that *Gambling Frequency* in our study is self-reported, and it refers specifically to *current* gambling habits. In contrast, the custom-screening of participants on Prolific was accomplished by using self-reported experience with one or more online gambling games without reference to recency of play. Indeed, some online gambling experience participants reported that they did not *currently* gamble when responding to the question about current *Gambling Frequency*. Our intended exploration of *Skill Gambler* participants was also complicated by the screener questions that may not be current in terms of gambling habits. Many individuals also reported experience with several of the listed games that included both skill-based games and games of chance, which limited the ability to identify gamblers who were more specialized in one type of game. Future research with more in-depth participant profile data can help resolve some of these concerns.

Another limitation of the study is the cross-sectional nature of the key participant characteristic data. In other words, it is difficult to establish a causal relationship between, for example, gambling frequency and probability judgment accuracy given that gambling frequency only varies across participants in our data set. While we interpret our findings to suggest that the frequency of one’s gambling impacts their judgment accuracy, we cannot say whether causation runs the other direction, or whether another unmeasured variable affects both—this criticism applied to most cross-sectional data studies. It is possible that those who poorly update probabilities do so in ways that promote overconfidence. This could then lead one to gamble more frequently, such that it is the approach to probability judgments that predict gambling frequency, as opposed to vice-versa.

Notwithstanding the limits of our data, the exploratory findings reported point to an interesting association between more gambling frequency and one’s approach to probability assessments. While all participants over-weighted low and under-weighted high base rate probabilities (as in Holt and Smith, 2009), and they conservatively incorporate new information into updating beliefs (as in Phillips and Edwards, 1966; Hill, 2017), more frequent gamblers were even more conservative, or “incomplete”, in their incorporation of new information into updating beliefs. This finding is noteworthy because we deliberately abstracted away from a risky choice task frequently encountered by gamblers or used in studies of gamblers to focus on a building block decision task that is of importance not only in gambling success, but also in the general domain of decision making under uncertainty.

Our results may be interpreted in light of others’ work on illusion of control among gamblers (Cowley et al., 2015). While our results cannot establish causation, as noted above, they are consistent with an illusion of control effect. Less accurate probability assessments do not improve one’s chance of gambling success, and so the fact that those least accurate in our Bayesian probability assessment task are those who gamble more frequently could point to an illusion of control at work in their gambling habits. Our task did not provide feedback on one’s accuracy across trials, and so our data show a snapshot view of how an individual approached the Bayesian inference task. In a gambling environment where feedback on success may stimulate learning, individuals may correct for faulty probability assessment efforts. Our data highlight that these more frequent gamblers may be less apt to learn from new information. We should note, however, that this speculation ignores the fact that confirmatory new information may be treated differentially compared to disconfirming information. An environment that embeds probability judgments within a task where judgment accuracy also implies additional benefits (i.e., increased chance of future gambling success) would help us more fully understand the implications suggested by our findings.

## Conclusion

This paper reported results from a pre-registered study of self-reported gambling patterns and decision making in an online incentivized Bayesian decision task environment. Such as previous research suggests, participants weighted all available information sources in their probability assessments (Grether, 1980). However, contrary to our hypotheses, we reported no significant differences in Bayesian accuracy between males and females, between problem and non-problem gamblers, nor between those with or without experience in skill-based gambling games. Consistent with this, we reported no differences in the same pairwise group comparisons regarding their approach to weighting base rate versus new information sources in forming probability assessments.

For our exploratory analysis, we found that those self-reporting more frequent gambling were less accurate in probability assessments, and those with higher scores on a cognitive reflection task were more accurate in their Bayesian accuracy. When examining these exploratory findings by participant sex, the link between frequent gambling and reduced Bayesian accuracy was significant only among females. However, the link between *CRT score* and increased accuracy was true for both male and female participants (if not a bit larger in magnitude in male participants). Corresponding findings from models estimating the weights placed on base rate versus sample evidence were consistent with the hypothesis that additional weight on new information is critical for more accurate probability assessments.

These exploratory findings suggest policy implications of interest. For example, if cognitive reflection aids in forming accurate probability assessments, then the profitability of the gambling industry depends (to some extent) on less reflective thinkers. While this is perhaps no surprise, it highlights a reason why Casinos promote alcohol consumption, engage emotion, or induce cognitive overload and fatigue—such items may reduce one’s tendency to engage in reflective and deliberative thinking. Or, if gambling frequency disproportionately harms probability judgments in females, then efforts to market habitual or regular gambling opportunities to females may be more profitable than the same efforts directed towards males. Of course, there is a degree of speculation in these suggested policy implications. However, they derive from the logic that, while the “house always wins”, it wins even more to the extent that gamblers cannot accurately assess the game’s uncertainty or the probability of winning versus losing.^10^ We leave it to future research to more systematically examine the importance of key gambler characteristics on the ability to assess and update probability judgments, and to provide complementary evidence regarding these intriguing exploratory findings and their implications.

## Supplementary Information

Below is the link to the electronic supplementary material.

Supplementary file1 (DOCX 31 KB)

Supplementary file2 (DOCX 131 KB)

## Data Availability

The data will be made available on the Open Science Framework link associated with the preregistration of this project upon publication.

## Footnotes

1 Also known as Bayes Theorem (Bayes, 1958), this rule describes conditional probabilities as a precise mathematical combination of new information and base rate probabilities. As such, it provides a mathematical foundation for reasoning out probability assessments in decision makers (Stone, 2013).

2 Note: the numbering or our hypotheses here differs from the preregistration plan for ease of exposition, but otherwise the hypotheses are unchanged.

3 See also Phillips and Edwards (1966) for an earlier version of a similar task referred to as the “Beads Task”.

4 Here, one holds a subjective belief regarding the actual event probability under consideration, *p.* This subject belief or weighted probability, $w\left(p\right)$, is defined as follows:

1 $$w\left(p\right)=\frac{{p}^{\gamma }}{{\left({p}^{\gamma }+{\left(1-p\right)}^{\gamma }\right)}^{1-\gamma }}$$

The weighting parameter, *γ*, equates subjective and objective (Bayesian) probabilities when *γ*= 1, but with *γ* < 1 the individual over-weights low and under-weights high probabilities. The odds ratio for one’s subjective probability estimate is therefore:

2 $$\frac{w(p)}{1-w(p)}=\frac{{p}^{\gamma }}{{\left({p}^{\gamma }+{\left(1-p\right)}^{\gamma }\right)}^{1-\gamma }}\bullet \frac{{\left((1-{p)}^{\gamma }-{\left((1-(1-p\right))}^{\gamma }\right)}^{1-\gamma }}{{\left(1-p\right)}^{\gamma }}={\left(\frac{p}{\left(1-p\right)}\right)}^{\gamma }$$

The typical odds ratio form of Bayes Rule then writes this subjective (posterior) odds ratio of the LEFT box being used as a function of the base rate odds ratio of LEFT (*Prob*_*LEFT*_) and the likelihood ratio of LEFT (i.e., the likelihood of a particular sample of evidence or new information, *S*, given one state of the world (LEFT box) versus the other (RIGHT box)). Thus, the odds ratio form of Bayes Rule in the context of our task is written as:

3 $$\frac{{Belief}_{LEFT}}{1-{Belief}_{LEFT}}={\left(\frac{{Prob}_{LEFT}}{\left(1-{Prob}_{LEFT}\right)}\right)}^{\gamma }\bullet {\left(\frac{Prob(S|LEFT)}{Prob\left(S|RIGHT\right)}\right)}^{\gamma }$$

5 Sleepiness was assessed given previous research connecting sleepiness and decision-making differences in this Bayesian choice task (e.g., Dickinson and Drummond, 2008; Dickinson et al., 2016).

6 The Prolific screener question is rather non-specific by asking about the types of online gambling games they have played, whereas the *Gambling Frequency* question specifically asked about *current* gambling. Thus, our *Gambling Frequency* variable will better distinguish current gamblers from non-gamblers in our data.

7 Our survey did not assess the proportion of one’s gambling that involved games of chance versus skill-based games. An alternative coding of *Skill Gambler* was explored where one was considered a *Skill Gambler* if they *only* reported playing skill-based games, as opposed to some mix of skill-based games and games of chance. Unfortunately, under that alternative coding, we have only n=20 such exclusive skill-based gamblers (and only 12 of those are non-problem gambles based on the NODS-CLiP* gambling screener administered).

8 Earlier work with this Bayesian updating task did not elicit probability estimates but participants were asked to merely indicate which box they deemed more likely in each trial. This approach lends itself to non-linear probit estimation of a binary variable indicating one’s subjective view of the more likely box, as a function of the odds and evidence. Here, we rescored the subjective probability estimate data to generate the binary indicator *Left Box Likely* = 1 if one’s subjective probability estimate of the trial using the LEFT box was greater than 50 (chances out of 100). Non-linear Probit models were then estimated to compare to the model estimates derived from the subjective log-odds estimates. These Probit estimations results are in Appendix A Table A3 and would compare to Appendix A Table A4. While the Probit estimations fail to fully utilize the available information in elicited responses, they nevertheless show consistency of findings. Specifically, both the odds and the evidence predict an increased probability of considering the *LEFT* box as more likely, and more frequent gamblers place a marginally lower weight on the new sample evidence (though with reduced statistical significance, *p* <.10).

9 See also Oechssler et al. (2009) for evidence that higher CRT improves one’s accuracy in probability updating.

10 Of course, this ignores other factors such as overconfidence and emotion, but the point is to highlight that probability judgments themselves are a key element to one’s decision to gamble or continue to gamble.
