# Supplementary material for: Gambling habits and Probability Judgements in a Bayesian Task Environment
Source: J Gambl Stud. 2024 Aug 27;40(4):2055–75. doi: 10.1007/s10899-024-10339-x (PMC11557619; doi:10.1007/s10899-024-10339-x)
Supplement: Supplementary file 1 — Supplementary file1 (DOCX 31 KB) [file 10899_2024_10339_MOESM1_ESM.docx]

**Appendix A (additional results)**

**TABLE A1:** Correlation matrix of key variables

|  | *Gfreq* | *ProbGamble* | *SkillGambler* | *SkillOnly* | *Female* | *Average RT* | *CRT score* |
| --- | --- | --- | --- | --- | --- | --- | --- |
| *Gfreq* | 1.000 |  |  |  |  |  |  |
| *ProbGamble* | 0.445 | 1.000 |  |  |  |  |  |
| *SkillGambler* | 0.710 | 0.364 | 1.000 |  |  |  |  |
| *SkillOnly* | 0.202 | 0.111 | 0.230 | 1.000 |  |  |  |
| *Female* | -0.099 | -0.104 | -0.090 | -0.083 | 1.000 |  |  |
| *Average RT* | -0.035 | 0.068 | -0.055 | -0.051 | -0.103 | 1.000 |  |
| *CRT score* | -0.001 | 0.008 | 0.021 | 0.013 | -0.268 | 0.104 | 1.000 |

**Notes**: *Gfreq* ∈ [0,4] describes self-reported *current* gambling frequency (0= never or non-gambler, higher values indicate more frequent online gambling). *ProbGamble* is an indicator = 1 if the respondent identified experienced any one or more of the 3 characteristics of problem gambling from the NODS rapid screener for adult pathological gambling. *SkillGamber* is an indicator = 1 if the individual indicated having played online gambling games of skill, while *SkillOnly* = 1 if the individual *exclusively* played games of skill (and no games of chance). *Female* = 1 that denotes sex (assigned at birth). *Average RT* is the average response time (in seconds) to the probability elicitation across the 20 trials in the Bayesian task. *CRT score* ∈ [1,6] is one’s score on the 6- item cognitive reflection task (higher scores indicating a more reflective style of thinking)

**TABLE A2:** Examining the important of current *Gambling Frequency* (versus Skill-game gambler)

| Dependent Variable:  ***Ln(Subjective Odds ratio)_Left_*** | (1) | (2) | (3) |
| --- | --- | --- | --- |
| **Variable** | Coef (st. error) | Coef (st. error) | Coef (st. error) |
| Constant | .044 (.025) | .023 (.045) | -.200 (.155) |
| *Ln(Prior Odds ratio)_Left_* | .318 (.036)** | .318 (.036)** | .318 (.036)** |
| *Ln(Likelihood ratio)_Left_* | .335 (.019)** | .334 (.019)** | .334 (.019)** |
| *Skill Gambler* (=1) | -.047 (.053) | -.049 (.053) | -.045 ((.051) |
| *Skill Gambler **  *Ln(Prior Odds ratio)_Left_* | -.083 (.078) | -.084 (.078) | -.084 (.078) |
| *Skill Gambler **  *Ln(Likelihood ratio)_Left_* | -.026 (.045) | -.025 (.045) | -.025 (.045) |
| *Gambling Frequency **  *Ln(Prior Odds ratio)_Left_* | .046 (.039) | .047 (.039) | .047 (.039) |
| *Gambling Frequency **  *Ln(Likelihood ratio)_Left_* | -.042 (.019)* | -.041 (.019)* | -.041 (.019)* |
| *Trial #* | --- | .004 (.003) | .004 (.003) |
| *Response Time* | --- | -.001 (.001) | -.001 (.001) |
| Age | --- | --- | .003 (.003) |
| Female (=1) | --- | --- | .022 (.040) |
| *Gambling Frequency* ∈ [0,4] | .0003 (.025) | .0004 (.025) | -.004 (.024) |
| *Prior Week Sleep Level* | --- | --- | .030 (.015)* |
| *Karolinska sleepiness* | --- | --- | -.010 (.010) |
| *CRT score* ∈ [0,6] | --- | --- | -.019 (.009)* |
| R-squared | .1031 | .1034 | .1045 |

**Notes:** **p* < .05, ***p* < 01 for the 1-tailed test of a pre-registered one-sided hypothesis (otherwise, *p-value* is for the 2-tailed test). *N*=9300 observations (standard errors adjusted for clustering at the participant level: n=465 clusters).

**TABLE A3:** Non-linear Probit model estimations examining the important of current *Gambling Frequency* (versus Skill-game gambler)—Marginal effects reported

| Dependent Variable:  ***Left Box likely* (=1)** | (1) | (2) | (3) |
| --- | --- | --- | --- |
| **Variable** | Coef (st. error) | Coef (st. error) | Coef (st. error) |
| *Ln(Prior Odds ratio)_Left_* | .024 (.012 )* | .024 (.012)* | .024 (.012)* |
| *Ln(Likelihood ratio)_Left_* | .052 (.005)** | .051 (.005)** | .051 (.005)** |
| *Skill Gambler* (=1) | -.0003 (.015) | -.001 (.015) | .003 (.015) |
| *Skill Gambler **  *Ln(Prior Odds ratio)_Left_* | -.014 (.027) | -.014 (.027) | -.014 (.027) |
| *Skill Gambler **  *Ln(Likelihood ratio)_Left_* | -.008 (.011) | -.008 (.011) | -.007 (.011) |
| *Gambling Frequency **  *Ln(Prior Odds ratio)_Left_* | .013 (.012) | .013 (.012) | .013 (.012) |
| *Gambling Frequency **  *Ln(Likelihood ratio)_Left_* | -.009 (.005)^ | -.009 (.005)^ | -.009 (.005)^ |
| *Trial #* | --- | .001 (.001) | .001 (.001) |
| *Response Time* | --- | -.001 (.0003)* | -.001 (.0003)* |
| Age | --- | --- | .002 (.001)* |
| Female (=1) | --- | --- | .005 (.011) |
| *Gambling Frequency* ∈ [0,4] | -.007 (.006) | -.007 (.006) | -.009 (.007) |
| *Prior Week Sleep Level* | --- | --- | .012 (.004)** |
| *Karolinska sleepiness* | --- | --- | .001 (.003) |
| *CRT score* ∈ [1,6] | --- | --- | -.004 (.003) |
| Psuedo R-squared | .0380 | .0386 | .0397 |

**Notes:** ^*p* < .10*,* **p* < .05, ***p* < 01 for the 1-tailed test of a pre-registered one-sided hypothesis (otherwise, *p-value* is for the 2-tailed test). *N*=9074 observations (standard errors adjusted for clustering at the participant level: n=465 clusters).

**TABLE A4:** Bayesian *Accuracy* and the impact of *Gambling Frequency* and *CRT Score*

| Dependent Variable:  ***Accuracy*** (trial level) | **Full Sample**  (1) | **Non-problem gamblers**  (2) |
| --- | --- | --- |
| **Variable** | Coef (st. error) | Coef (st. error) |
| Constant | .631 (.066)** | .597 (.132)** |
| Female (=1) | .020 (.030) | .060 (.083) |
| *Trial #* | .0003 (.0004) | -.001 (.001) |
| *Response Time* | .0002 (.0002) | .0005 (.0004) |
| Age | -.0002 (.001) | -.002 (.002) |
| *Prior Week Sleep Level* | .006 (.005) | .020 (.012) |
| *Karolinska sleepiness* | .002 (.004) | .003 (.008) |
| *Skill Gambler* (=1) | -.009 (.020) | -.034 (.036) |
| Gambling Frequency ∈ [0,4] | -.013 (.011) | -.014 (.021) |
| *CRT score* ∈ [0,6] | .017 ( 005)** | .032 (.022)* |
| *Gambling Frequency * Female* | -.025 (.014) | -.059 (.033) |
| *CRT score * Female* | -.003 (.627) | .001 (.013) |
| **F-Test**: *Gambling Freq + Gambling Freq * Female = 0* | *F* (1,464) = 8.81  *P* = .0032 | *F* (1,128) = 8.29  *P* = .0047 |
| **F-Test:** *CRT Score + CRT Score * Female = 0* | *F* (1,464) = 8.58  *P* = .0036 | *F* (1,128) = 8.82  *P* = .0036 |
| Number of observations (participant clusters) | 9300 (465 participants) | 2580 (129 participants) |
| R-squared | .0368 | .0673 |

**Notes:** ^*p* < .10*,* **p* < .05, ***p* < 01 for the 2-tailed test). Standard errors adjusted for clustering at the participant level. Figure 2 and 3 in the main text show results from the full sample model (1) coefficient estimates and their interaction terms.
