# Supplementary material for: Gambling habits and Probability Judgements in a Bayesian Task Environment
Source: J Gambl Stud. 2024 Aug 27;40(4):2055–75. doi: 10.1007/s10899-024-10339-x (PMC11557619; doi:10.1007/s10899-024-10339-x)
Supplement: Supplementary file 2 — Supplementary file2 (DOCX 131 KB) [file 10899_2024_10339_MOESM2_ESM.docx]

**Appendix B (complete survey)**

**Informed Consent:** You are being asked to complete this online survey as part of a research study on decision making related dietary choice. Participation in this online survey is completely voluntary, your responses to this survey will remain completely confidential, the data will be securely stored, your name will not be recorded anywhere on this survey. The only identifier we will record will be your Prolific ID, which we as researchers cannot link to personally identifiable data of yours. This survey is estimated to take 18 minutes to complete and your payment for successful and complete survey completion will be $2.40.  Additionally, the information use decision task within this survey offers **the chance of earning an *additional* $1.00 bonus payment,** depending on your choice in the task (the instructions will clearly explain how this works on that task). There are no known risks associated with this study beyond those associated with everyday life. Although this study will not benefit you personally, its results will help our understanding of how people make decisions.

 For additional information related to this questionnaire, contact Dr. David Dickinson, Department of Economics, Appalachian State University, at dickinsondl@appstate.edu. Appalachian State University's Institutional Review Board (IRB) has determined this study to be exempt from review by the IRB administration**.**

- **I Consent** and wish to continue with this study
- **I do not consent** to participating and **do not wish to continue**

| Page Break |  |
| --- | --- |

As you do not wish to participate in this study, please return your submission on Prolific by selecting the 'Stop without completing' button

| Page Break |  |
| --- | --- |
|  |  |

The following questions are **screener validation questions** to make sure we get the desired sample we advertised for this survey

| Page Break |  |
| --- | --- |

What is **your current age** (in years)?

|  | 18 | 26 | 34 | 43 | 51 | 59 | 67 | 75 | 84 | 92 | 100 |
| --- | --- | --- | --- | --- | --- | --- | --- | --- | --- | --- | --- |

| Years of age () | 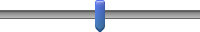 |
| --- | --- |

**What is your sex?**
(i.e., what sex were you assigned at birth, such as on an original birth certificate)?

- Female (1)
- Male (2)

**What types of online gambling / casino games have you played?** Choose all that apply.

- Baccarat
- BlackJack)
- Bingo)
- Craps)
- Lottery)
- Pachinko
- Poker)
- Race & Sports Book
- Roulette1)
- Slots
- Video Poker
- Virtual Sports Betting
- None of the above
- Not applicable / rather not say

**What is your current frequency of gambling** (online or otherwise)?

- Never
- *less* than once a month
- once or twice a month
- once or twice a week
- daily

| Page Break |  |
| --- | --- |

**In what country do you currently reside?**

- United Kingdom
- United States
- Other

| Page Break |  |
| --- | --- |

Before you start, please switch off phone/ e-mail/ music so that you can focus on this study.  Thank you!

 Please carefully enter your Prolific ID

________________________________________________________________

Please mark the number that best corresponds to how sleepy you feel **right now**. You may mark any number, but mark only one number.

- 1. Extremely alert
- 2.
- 3. Alert
- 4.
- 5. Neither alert nor sleepy
- 6.
- 7. Sleepy--but no difficulty remaining awake
- 8.
- 9. Extremely sleepy--fighting sleep

| Page Break |  |
| --- | --- |

**Over the last 7 nights**, what is the average amount of sleep you obtained each night?

|  | 0 | 1 | 2 | 3 | 4 | 5 | 6 | 7 | 8 | 9 | 10 | 11 | 12 |
| --- | --- | --- | --- | --- | --- | --- | --- | --- | --- | --- | --- | --- | --- |

| Average nightly sleep over the LAST WEEK () | 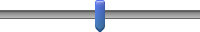 |
| --- | --- |

| Page Break |  |
| --- | --- |

**Last night**, how much sleep did you get?

|  | 0 | 1 | 2 | 3 | 4 | 5 | 6 | 7 | 8 | 9 | 10 | 11 | 12 |
| --- | --- | --- | --- | --- | --- | --- | --- | --- | --- | --- | --- | --- | --- |

| Hours of sleep LAST NIGHT () | 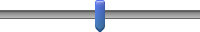 |
| --- | --- |

| Page Break |  |
| --- | --- |

Q3.5 **What do you feel is the optimal amount of sleep for you personally to get each night**? (optimal in terms of next day alertness, performance, and functionality for you personally.)

|  | 0 | 1 | 2 | 3 | 4 | 5 | 6 | 7 | 8 | 9 | 10 | 11 | 12 |
| --- | --- | --- | --- | --- | --- | --- | --- | --- | --- | --- | --- | --- | --- |

| Average nightly sleep I need personally () | 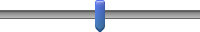 |
| --- | --- |

| Page Break |  |
| --- | --- |

Have there ever been periods lasting 2 weeks or longer when you spent a lot of time thinking about your gambling experiences or planning out future gambling ventures or bets?

- NO
- YES

|  |  |
| --- | --- |
|  |  |
| Page Break |  |

Have you ever tried to stop, cut down, or control your gambling?

- NO
- YES

| Page Break |  |
| --- | --- |

Have you ever lied to family members, friends, or others about how much you gamble or how much money you lost on gambling?

- NO
- YES

| Page Break |  |
| --- | --- |

As described earlier, we are interested in factors that influence the decisions you might make. In order for the results of this survey to be valid, **it is essential that you read all the instructions and questions carefully**. So we know that you have read these instructions, please place the slider below on the answer to (33+12)=? Thank you for taking the time to read these instructions.

|  | 0 | 10 | 20 | 30 | 40 | 50 | 60 | 70 | 80 | 90 | 100 |
| --- | --- | --- | --- | --- | --- | --- | --- | --- | --- | --- | --- |

| My response () | 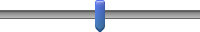 |
| --- | --- |

| Page Break |  |
| --- | --- |

**INSTRUCTIONS FOR THE DECISION TASK**
 
In each round, you will see a picture of two boxes, populated with 3 total balls each.  The LEFT box contains 2 black and 1 white ball, while the RIGHT box contains 2 white and 1 black ball.  One of the boxes will be selected in each round.  You will not know for certain which box is selected, but **we will provide you with two pieces of information that may be helpful in how you determine which box was more likely selected**.  First, we will give you the "starting chances" that either box may be selected in that round.  Higher starting chances of the LEFT box means it is more likely the LEFT box will be selected in that round, for example. Secondly, we will present to you the results of having drawn 8 balls, with replacement, from whichever box was selected.  Drawing with replacement means we always replace the ball after drawing so that the contents of each box are always the same when making each draw.  All else equal, drawing balls from the LEFT box (which has more black than white balls) is more likely to produce more black balls in the sample set of draws, and drawing balls from the RIGHT box (which has more white than black balls) is more likely to produce more white balls in a sample of draws.  Thus, both the "starting chances" and the "sample evidence" may be useful as you think of which box had more likely been selected in that round.
  
Winning a $0.05 bonus in each round depends on your response in that round. At the most basic level, in each round **the goal is to give your best guess about how likely *the LEFT box* was selected in that round.  We will ask for your answer each round by asking you for your best estimate of the "*chances out of 100*" that you think the LEFT box was selected in that round**.  A "0" answer means you feel there was no chance the LEFT box was selected in that round, "50" means you feel there was an equal chance the LEFT or RIGHT box was selected that round, and "100" means you feel that the LEFT box was certainly selected in that round.  **Because we are asking for your response in terms of *how likely you think it is the LEFT box was selected*, you should indicate a response greater than "50" if you feel it is more likely the LEFT box was selected in that round, and a response less than "50" if you feel it is more likely the RIGHT box was selected (and the closer to 100 or 0 your response, the more strongly you feel the box selected was the LEFT or RIGHT, respectively).** The payment method for this task is designed so that your chances of winning a bonus that round are highest if your response is an accurate reflection of how likely you think the LEFT box was selected in that round.  You will maximize your chance of the highest bonus in this decision task by being as accurate as possible in each round.

| Page Break |  |
| --- | --- |

**Here's how your response generates a bonus in each round of this task.** It is not the most easy to understand process, but its design actually ensures that it is in your best interest (in terms of bonus payment potential) to response with your true belief in each round.

 (you can **skip these shaded details if you are not interested in the underlying process**).

 In each round, the computer will draw a random number from 0 to 100. Each number from 0 to 100 is equally likely to be drawn by the computer. We'll call this number Draw 1. How you win or lose that round of the task depends on what number the computer draws for Draw 1 and your belief response (in terms of the "chances out of 100" that you think the LEFT box was selected in that round):

 *Payment Method 1)*If Draw 1 is less than your belief response, you win if the LEFT box was selected and you do not win if the RIGHT box was selected in that round. For example, if you enter a belief response of 99, you are very likely to win a bonus if the LEFT box was selected and very likely to not win if the RIGHT box was selected. You are more likely to win the bonus in any given round where the LEFT box was selected if you give a higher belief response (i.e., chances out of 100 you feel the LEFT box was selected).  Similarly, if the RIGHT box was selected in any given round, your are more likely to win the *lower* is your belief response regarding how likely the LEFT box was selected (because if indicate you feel it was less likely the LEFT box was selected, then you are also indicating that you feel it more likely the RIGHT box was selected in that round). 

 *Payment Method 2*) If Draw 1 is greater than your response, then the computer will draw a second random number from 0 to 100. As before, each number from 0 to 100 is equally likely to be drawn by the computer. We'll call this random number Draw 2. If Draw 2 is less than Draw 1, then you win the bonus for that round. If Draw 2 is greater than Draw 1, then you do not win the round.  What *Payment Method 2*does is provide a way where, on average, you have a higher chance of winning the bonus than with *Payment Method 1* whenever Draw 1 is greater than your response. 
 
The computer will therefore use your response each round to choose the whichever payoff method (Method 1 or Method 2) that offers you the best chance of earning the bonus payment in that round.  **If your response represents your true beliefs about the chances the LEFT box was selected, then the computer selects the payment method that gives you the best chance of winning the bonus that round.**

| Page Break |  |
| --- | --- |

**Instructions (continued)**

Again, the contest payment process is designed so that you have the best chance for earning a 5 cent bonus each round by being as accurate as possible with your response (which can earn you up to a total bonus payment of 20 rounds times 5 cents, or $1.00). The random numbers and payment calculations will happen behind the scenes after you have finished the study. As such, you will not have any feedback on your performance from one round to the next (you will only know your outcome based on the bonus you receive separately from the fixed payment for this task) 


 A picture of the stimulus is shown below, which succinctly reminds you of the contents of the LEFT and RIGHT boxes (this remains constant across all trials), as well as the starting-chance of selecting the LEFT versus RIGHT box and the sample evidence of the 8 ball drawn from the selected box. Across different rounds, the starting-chance and/or sample evidence may change, and so you should pay attention to these pieces of information carefully in each round because this may affect how likely you think it was that the LEFT box was selected in that round. You may use the starting-chance and sample evidence information however you like in giving your best estimate of the "chances out of 100" the LEFT box was selected in that round, and remember that you maximize your chance of the highest bonus by responding what you truly believe the chances are that the LEFT box was selected in each and every round.

| Page Break |  |
| --- | --- |


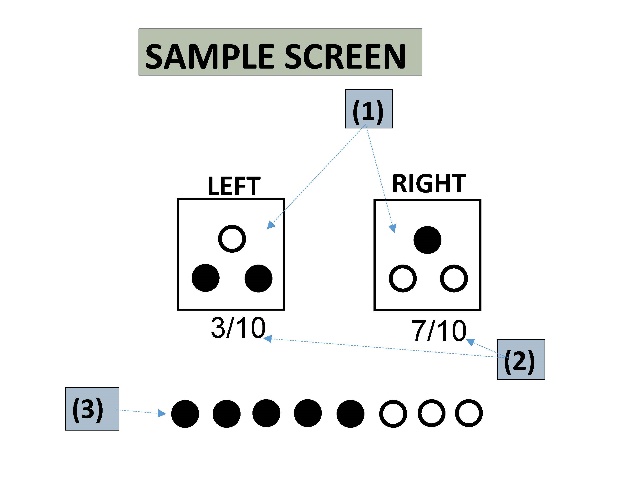

 **The importance of each part of the stimulus image is as follows:**
 
**(1)**  Balls inside of the box show the different contents of each box
 
**(2)**  The fraction beneath the box shows the starting-chance (out of 10) that the box will be selected.  ***A greater fraction below the LEFT box means the starting-chance of selecting the LEFT box is higher*** *(and a lower fraction means the starting-chance of selecting the LEFT box is lower)*.  However, remember that you do **not** get to see which Box was actually selected
 
**(3)**  The set of 8 balls at the bottom show the result of drawing 8 balls, with replacement, from the Box that was selected.  Remember, because of the contents of each box shown in **(1)**, ***a sample draw with more black balls is more likely to come from the LEFT box*** *(and a draw with more white balls is less likely to come from the LEFT box).*
 
 
 
   Using any of this information that seems relevant to you, **you are then asked to indicate the likelihood (chances out of 100) that you think the LEFT box had been selected** in that particular trial.
 
**Note:** From trial to trial, *the information in items (2) and (3) may change* (but not item (1)--the contents of each Box).
 
**Remember, you maximize the chance of winning the bonus payment each round by responding with your true belief of how likely you think the LEFT box was selected, given the available information!**

| Page Break |  |
| --- | --- |

The main assessment task starts on the next page.  Please click below when ready to start.

- I'm ready to start the task

| Page Break |  |
| --- | --- |

**NOTE: 2 sample trials are shown on the following pages for purposes of this Appendix. The complete survey included 20 trials where the details on each trial varied the number of black versus white balls in the 8-sample draw, and/or vary the fractions beneath the LEFT and RIGHT boxes (See Table 1, main text).**

Please indicate on the scale below **how likely you think it is that the LEFT box had been selected**, given the following information below:
 
(remember, the fractions listed directly below each box indicate the starting-chance that the box will be selected in this trial.  The row of 8 balls underneath show the result of drawing 8 balls with replacement from the box actually selected in this trial).  
   

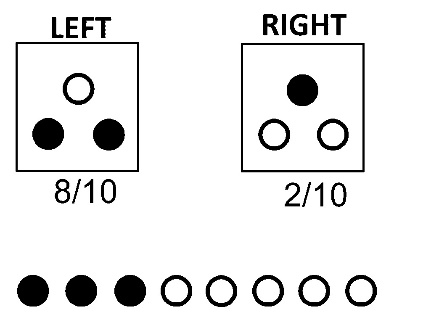

 
Given this information, I feel the chances out of 100 (i.e., the likelihood) that the LEFT box was selected in this trial is:

|  | Low chances out of 100 mean I think it is **unlikely the LEFT box was selected** (i.e., more likely the RIGHT box was selected) | Chance of 50 out of 100 means I think it is **equally likely that either the LEFT or RIGHT box were selected** | High chances out of 100 mean I think it is**more likely the LEFT box was selected** (i.e., unlikely the RIGHT box was selected) |
| --- | --- | --- | --- |

|  | 0 | 10 | 20 | 30 | 40 | 50 | 60 | 70 | 80 | 90 | 100 |
| --- | --- | --- | --- | --- | --- | --- | --- | --- | --- | --- | --- |

| Chances out of 100 the **LEFT** box was selected in this trial. () | 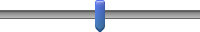 |
| --- | --- |

| Page Break |  |
| --- | --- |

Please indicate on the scale below **how likely you think it is that the LEFT box had been selected**, given the following information below:
 
(remember, the fractions listed directly below each box indicate the starting-chance that the box will be selected in this trial.  The row of 8 balls underneath show the result of drawing 8 balls with replacement from the box actually selected in this trial).  
 
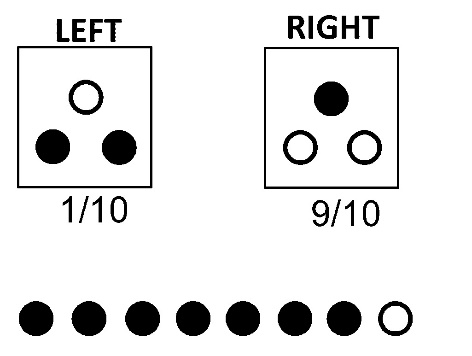

 
 
 Given this information, I feel the chances out of 100 (i.e., the likelihood) that the LEFT box was selected in this trial is:

|  | Low chances out of 100 mean I think it is **unlikely the LEFT box was selected** (i.e., more likely the RIGHT box was selected) | Chance of 50 out of 100 means I think it is **equally likely that either the LEFT or RIGHT box were selected** | High chances out of 100 mean I think it is**more likely the LEFT box was selected** (i.e., unlikely the RIGHT box was selected) |
| --- | --- | --- | --- |

|  | 0 | 10 | 20 | 30 | 40 | 50 | 60 | 70 | 80 | 90 | 100 |
| --- | --- | --- | --- | --- | --- | --- | --- | --- | --- | --- | --- |

| Chances out of 100 the **LEFT** box was selected in this trial. () | 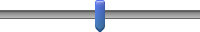 |
| --- | --- |

| Page Break |  |
| --- | --- |

Finally, please answer these final questions on the next set of pages for us.

| Page Break |  |
| --- | --- |

A bat and a ball cost $1.10 in total.  The bat costs $1.00 more than the ball.  How much does the ball cost?
(please indicate your numeric answer **in cents**)

________________________________________________________________

| Page Break |  |
| --- | --- |

If it takes 5 minutes for 5 machines to make 5 widgets, how long would it take for 100 machines to make 100 widgets?
(please indicate your numeric answer **in minutes)**

________________________________________________________________

| Page Break |  |
| --- | --- |

If 3 elves can wrap 3 toys in 1 hour, how many elves are needed to wrap 6 toys in 2 hours?
(please give your numeric answer in **# of elves**)

________________________________________________________________

| Page Break |  |
| --- | --- |

Jerry received both the 15th highest and the 15th lowest mark in the class. How many students are there in the class?
(please give your numeric answer in **# of students)**

________________________________________________________________

| Page Break |  |
| --- | --- |

In an athletics team, tall members are **three** times more likely to win a medal than short members. This year the team has won 60 medals so far. How many of these have been won by short athletes?
(please give your numeric answer in **# of medals**)

________________________________________________________________

| Page Break |  |
| --- | --- |

In a lake, there is a patch of lily pads. Every day, the patch doubles in size. If it takes 48 days for the patch to cover the entire lake, how long would it take for the patch to cover **half**of the lake? (please indicate your numeric answer **in days**)

________________________________________________________________
